# Supplementary material for: Pulmonary surfactant inhibition of nanoparticle uptake by alveolar epithelial cells
Source: Sci Rep. 2020 Nov 10;10:19436. doi: 10.1038/s41598-020-76332-7 (PMC7655959; doi:10.1038/s41598-020-76332-7)
Supplement: Supplementary file 1 — Supplementary Information. [file 41598_2020_76332_MOESM1_ESM.docx]

Supplementary Information

Pulmonary surfactant inhibition of nanoparticle uptake by alveolar epithelial cells

**M. Radiom^1,2*,^, M. Sarkis^1^, O. Brookes^3^, E.K. Oikonomou^1^, A. Baeza-Squiban^3^ and J.-F. Berret^1*^**

*^1^Université de Paris, UMR CNRS 7057, Laboratoire Matière et Systèmes Complexes, Paris, France.*

*^2^Present address: Institute for Food, Nutrition and Health, D-HEST, ETH Zürich, Zürich, Switzerland*

*^3^Université de Paris Unité BFA, UMR CNRS 8251, Laboratoire de Réponses Moléculaires et Cellulaires aux Xénobiotiques, Paris, France*

Correspondence: [milad.radiom@hest.ethz.ch](mailto:milad.radiom@hest.ethz.ch), [jean-francois.berret@u-paris.fr](mailto:jean-francois.berret@univ-paris-diderot.fr)

**Outline**

**S1** – Production volume and industrial applications for silica nanoparticles (2012)

**S2** – Curosurf composition and comparison with native surfactant

**S3** – Summary of exposure conditions on glass coverslip

**S4** – Summary of exposure conditions on transwell

**S5** – Cryo-TEM images of silica nanoparticles

**S6** – Dynamic light scattering of silica nanoparticles dispersion in culture media

**S7** – Cryo-TEM images of Curosurf vesicles

**S8** – Dynamic light scattering of silica nanoparticle-Curosurf dispersions

**S9** – Cellular viability assays

**S10** – TEM of internalized silica nanoparticles without or with Curosurf

**Supplementary Information S1**

Production volume and industrial applications for silica nanoparticles (2012)

List of silica nanoparticle applications^5^:

| **Silica nanoparticles**  Production volumes for nanomaterials (2012): 500 000 tons |
| --- |
| Aerogels as light-weight materials and thermal insulators |
| Filler in rubber and polymer compound materials |
| Nanoelectronics fabrication as mask substrate |
| Bioceramics (as coatings for implants) |
| Anti-corrosion and wear resistant coatings |
| Insulators with high dielectric properties |

**Supplementary Information S2**

Curosurf composition and comparison with native surfactant

Lipid and protein compositions of native surfactant obtained by saline bronchoalveolar lavage compared to that of Curosurf, a pulmonary surfactant substitute indicated for the rescue treatment of Respiratory Distress Syndrome (RDS) in premature infants^1^. The concentrations are given in percentage by weight of the total lipid content.

| **Lipid composition**  **(%, g/g of total lipid)** | **Native Surfactant** | **Curosurf** |
| --- | --- | --- |
| Phosphatidylcholine (PC) | 70 – 85 | 67 – 74 |
| Lysophosphatidylcholine (LPC) | 0.5 | < 1 |
| Sphingomyelin (SM) | 2 | 8.1 |
| Cholesterol | 5 | 0 |
| Phosphatidylinositol (PI) | 4 – 7 | 3.3 |
| Phosphatidylserine (PS) | 5 |  |
| Phosphatidylethanolamine (PE) | 3 | 4.5 |
| Phosphatidylglycerol (PG) | 7 – 10 | 1.2 |
| **Protein concentration**  **(%, g/g of total lipid)** | **Native Surfactant** | **Curosurf** |
| SP-A | 4 | 0 |
| SP-B | 1 | 0.3 |
| SP-C | 1 | 0.7 |
| SP-D | 4 | 0 |

**Supplementary Information S3**

Summary of exposure conditions on glass coverslip

Generally, four exposure conditions were used including neat nanoparticles, X = 2, X = 20 and neat Curosurf. The table below summarizes the dispersion preparations for exposure on coverslip. (Top) Concentration, volume and resulting mass of silica nanoparticles and Curosurf during sample preparation. (Middle) Volume and surface concentration of silica nanoparticles in the final dispersion. (Bottom) Volume and surface concentration of Curosurf in the final dispersion. Calculations are for coverslip cell culture support placed inside a 6-well plate, $=9.6 \mathrm{cm}^{2}$ . The difference $V_{TOT}-\left( V_{NP}+V_{Curo} \right)$ is made up by addition of serum free culture medium.

| Initial | Exposure | $c_{NP}$  (g l^-1^) | $V_{NP}$  (µl) | $m_{NP}$  (µg) | $c_{Curo}$  (g l^-1^) | $V_{Curo}$  (µl) | $m_{Curo}$  (µg) |
| --- | --- | --- | --- | --- | --- | --- | --- |
|  | Neat nanoparticle | 0.2 | 1000 | 200 |  |  | 0 |
|  | X = 2 | 4 | 50 | 200 | 4 | 100 | 400 |
|  | X = 20 | 21 | 9.5 | 199.5 | 21 | 190.5 | 4000.5 |
|  | Neat  Curosurf |  |  | 0 | 4 | 100 | 400 |
|  |  |  |  |  |  |  |  |

| Final – nanoparticle | Exposure | $m_{NP}$  (µg) | $V_{TOT}$  (µl) | $c_{NP}$  (g l^-1^) | $A$  (cm^2^) | ${m_{NP}}/A$  (µg cm^-2^) |
| --- | --- | --- | --- | --- | --- | --- |
|  | Neat nanoparticle | 200 | 2000 | 0.1 | 9.6 | 20.8 |
|  | X = 2 | 200 | 2000 | 0.1 | 9.6 | 20.8 |
|  | X = 20 | 199.5 | 2000 | 0.1 | 9.6 | 20.8 |
|  | Neat  Curosurf | 0 | 2000 | 0 | 9.6 | 0 |
|  |  |  |  |  |  |  |

| Final – Curosurf | Exposure | $m_{Curo}$  (µg) | $V_{TOT}$  (µl) | $c_{Curo}$  (g l^-1^) | $A$  (cm^2^) | ${m_{Curo}}/A$  (µg cm^-2^) |
| --- | --- | --- | --- | --- | --- | --- |
|  | Neat nanoparticle | 0 | 2000 | 0 | 9.6 | 0 |
|  | X = 2 | 400 | 2000 | 0.2 | 9.6 | 41.6 |
|  | X = 20 | 4000.5 | 2000 | 2 | 9.6 | 416 |
|  | Neat  Curosurf | 400 | 2000 | 0.2 | 9.6 | 41.6 |
|  |  |  |  |  |  |  |

**Supplementary Information S4**

Summary of exposure conditions on transwell

Generally, four exposure conditions were used including neat nanoparticles, X = 2, X = 20 and neat Curosurf. The table below summarizes the dispersion preparations for exposure on transwell. (Top) Concentration, volume and resulting mass of silica nanoparticles and Curosurf during sample preparation. (Middle) Volume and surface concentration of silica nanoparticles in the final dispersion. (Bottom) Volume and surface concentration of Curosurf in the final dispersion. Calculations are for transwell insert, $=1.12 \mathrm{cm}^{2}$ . The difference $V_{TOT}-\left( V_{NP}+V_{Curo} \right)$ is made up by addition of serum free culture medium.

| Initial | Exposure | $c_{NP}$  (g l^-1^) | $V_{NP}$  (µl) | $m_{NP}$  (µg) | $c_{Curo}$  (g l^-1^) | $V_{Curo}$  (µl) | $m_{Curo}$  (µg) |
| --- | --- | --- | --- | --- | --- | --- | --- |
|  | Neat nanoparticle | 0.2 | 250 | 50 |  |  | 0 |
|  | X = 2 | 4 | 12.5 | 50 | 4 | 25 | 100 |
|  | X = 20 | 21 | 2.4 | 50.4 | 21 | 47.6 | 999.6 |
|  | Neat  Curosurf |  |  | 0 | 4 | 25 | 100 |
|  |  |  |  |  |  |  |  |

| Final – nanoparticle | Exposure | $m_{NP}$  (µg) | $V_{TOT}$  (µl) | $c_{NP}$  (g l^-1^) | $A$  (cm^2^) | ${m_{NP}}/A$  (µg cm^-2^) |
| --- | --- | --- | --- | --- | --- | --- |
|  | Neat nanoparticle | 50 | 500 | 0.1 | 1.12 | 44.6 |
|  | X = 2 | 50 | 500 | 0.1 | 1.12 | 44.6 |
|  | X = 20 | 50.4 | 500 | 0.1 | 1.12 | 45.0 |
|  | Neat  Curosurf | 0 | 500 | 0 | 1.12 | 0 |
|  |  | $m_{NP}$  (µg) | $V_{TOT}$  (µL) | $c_{NP}$  (g L^-1^) |  |  |

| Final – Curosurf | Exposure | $m_{Curo}$  (µg) | $V_{TOT}$  (µl) | $c_{Curo}$  (g l^-1^) | $A$  (cm^2^) | ${m_{Curo}}/A$  (µg cm^-2^) |
| --- | --- | --- | --- | --- | --- | --- |
|  | Neat nanoparticle | 0 | 500 | 0 | 1.12 | 0 |
|  | X = 2 | 100 | 500 | 0.2 | 1.12 | 89.3 |
|  | X = 20 | 999.6 | 500 | 2 | 1.12 | 892.5 |
|  | Neat  Curosurf | 100 | 500 | 0.2 | 1.12 | 89.3 |
|  |  |  |  |  |  |  |

**Supplementary Information S5**

Cryo-TEM images of silica nanoparticles

Cryo-TEM images (a) and size distribution (b) of silica nanoparticles. The distribution is well accounted for by a log-normal function of median 41.2 nm and dispersity 0.11.

| 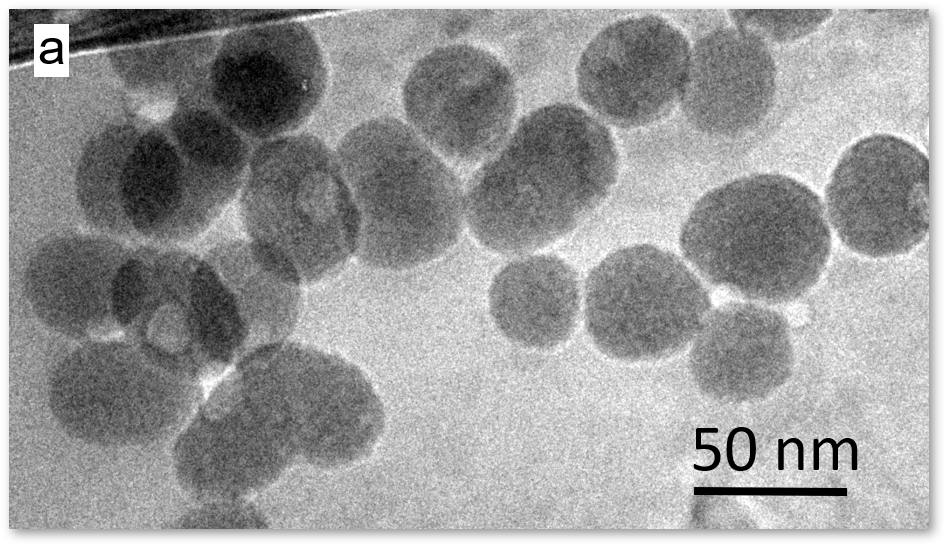 | 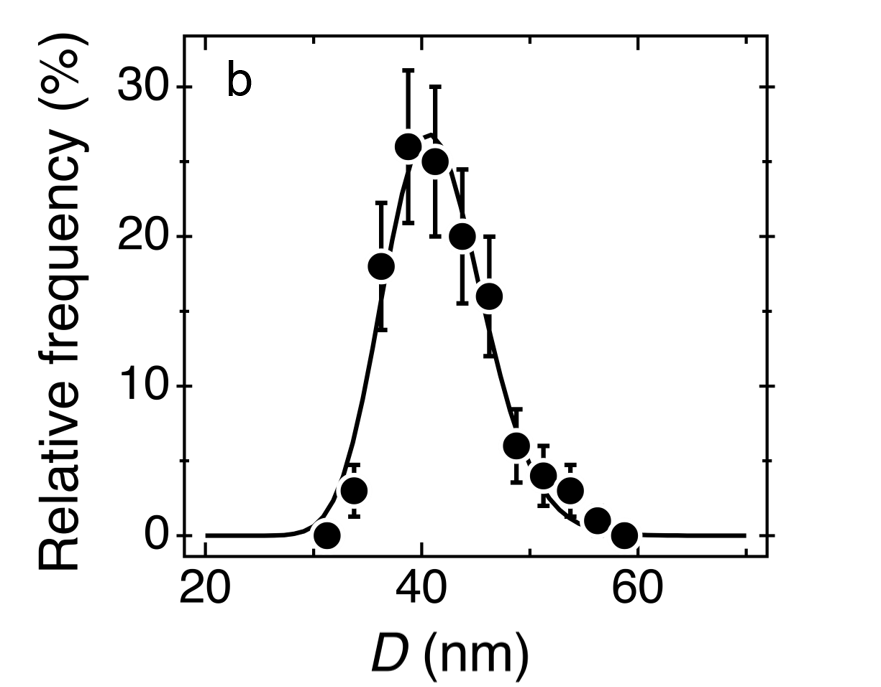 |
| --- | --- |

**Supplementary Information S6**

Dynamic light scattering of silica nanoparticles dispersion in culture media

Silica nanoparticles in DMEM and RPMI after 5-minute incubation (25^°^C). The DLS experiments were performed at two concentrations, 0.1 and 1 g l^-1^, both with and without fetal bovine serum (FBS).

| **Conc.**  **(g l^-1^)** | **Solvent** | **FBS** | **D_H_**  **(nm)** | **± Delta (D_H_)**  **(nm)** | **Derived**  **count rate**  **(kcps)** |
| --- | --- | --- | --- | --- | --- |
| 0.1 | DMEM | no | 1309 | 385 | 6089 |
| 0.1 | DMEM | yes | 380 | 124 | 25244 |
|  |  |  |  |  |  |
| 1 | DMEM | no | 6813 | 1580 | 31849 |
| 1 | DMEM | yes | 4181 | 1190 | 43258 |
|  |  |  |  |  |  |
| 0.1 | RPMI | no | 1196 | 328 | 6394 |
| 0.1 | RPMI | yes | 441 | 159 | 23194 |
|  |  |  |  |  |  |
| 1 | RPMI | no | 3863 | 1018 | 31487 |
| 1 | RPMI | yes | 3616 | 1140 | 42071 |
|  |  |  |  |  |  |

Silica nanoparticles in DMEM and RPMI after 120-minute incubation (25^°^C). The DLS experiments were performed at two concentrations, 0.1 and 1 g l^-1^, both with and without fetal bovine serum (FBS).

| **Conc.**  **(g l^-1^)** | **Solvent** | **FBS** | **D_H_**  **(nm)** | **± Delta (D_H_)**  **(nm)** | **Derived**  **count rate**  **(kcps)** |
| --- | --- | --- | --- | --- | --- |
| 0.1 | DMEM | no | 1235 | 274 | 5976 |
| 0.1 | DMEM | yes | 396 | 117 | 29120 |
|  |  |  |  |  |  |
| 1 | DMEM | no | 3099 | 869 | 37185 |
| 1 | DMEM | yes | 2704 | 788 | 47335 |
|  |  |  |  |  |  |
| 0.1 | RPMI | no | 1563 | 428 | 6121 |
| 0.1 | RPMI | yes | 467 | 151 | 28148 |
|  |  |  |  |  |  |
| 1 | RPMI | no | 2970 | 673 | 34970 |
| 1 | RPMI | yes | 2401 | 759 | 49447 |
|  |  |  |  |  |  |

**Supplementary Information S7**

Cryo-TEM images of Curosurf vesicles

Cryo-TEM images of Curosurf vesicles at a concentration of 5 g l^-1^ ^2-4^.


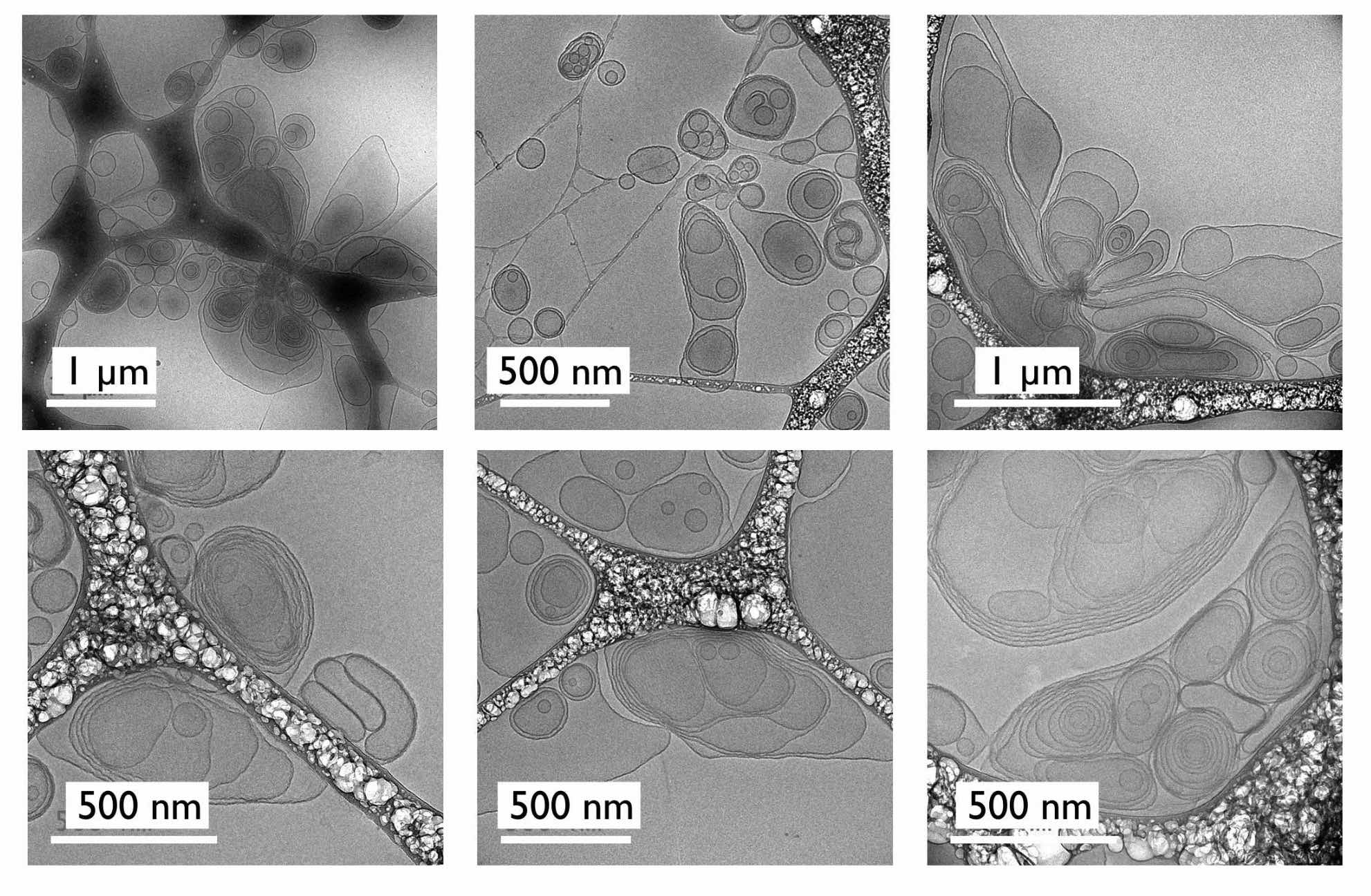


**Supplementary Information S8**

Dynamic light scattering of silica nanoparticles-Curosurf dispersions

Scattered intensity (a and c) and hydrodynamic diameter (b and d) of silica particles mixed with Curosurf at T = 25^º^C (a and b) and at T = 37^º^C (c and d). *X* is the ratio between the surfactant and nanoparticle mass concentrations. Continuous lines in green in a and c represent the scattered intensities calculated assuming that particles and vesicles do not interact. The error bars represent the mean of the standard deviations for measurements made in triplicate.


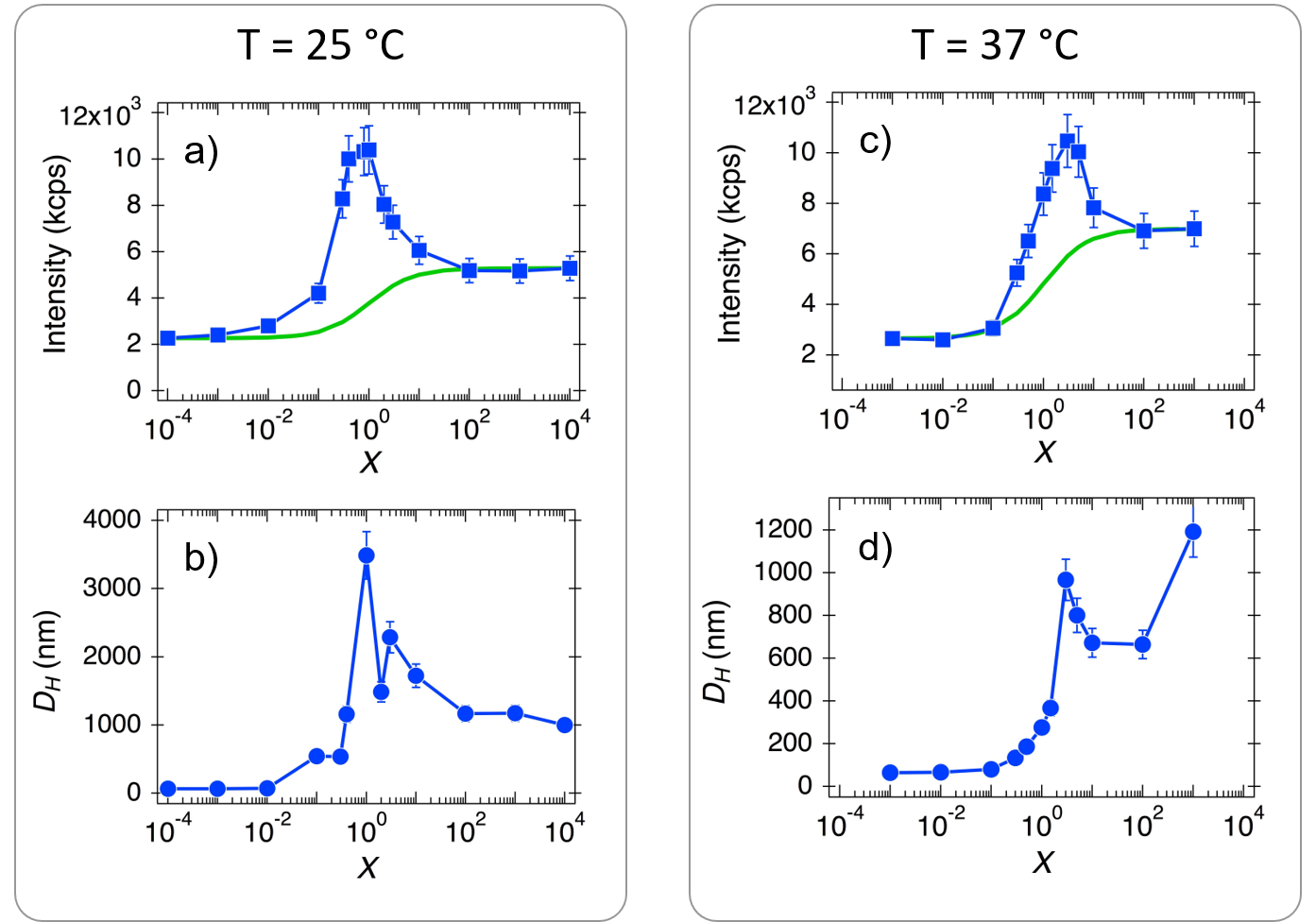


**Supplementary Information S9**

Cellular viability assays

*WST-1 toxicity assay.* Cells were seeded in a 96-well plate at 20k cells per well in white DMEM with serum. After 24 h, the cells were rinsed with PBS and incubated for another 24 h at 37°C with 200 μl of silica in white DMEM. The cells were rinsed with PBS and incubated for 30 min with 100 μl of WST-1 per well (dilution 1:20 in white DMEM). The assay is based upon the reduction by cellular dehydrogenase of the achromatic tetrazolium salt WST-1 to the dark yellow colored soluble formazan. The generation of formazan was measured at 450 nm in a microplate reader (ELx808, Biotek Instruments, Winooski, United States) against a blank containing culture media and WST-1 and the data were corrected from the absorbance at 630 nm. Positive controls were performed with hydrogen peroxide and the non-interference between nanoparticles, cells and WST-1 was assessed treating half of the cultures with Triton (2% in white DMEM, 15 min of incubation at 37°C). Data are represented as means ± standards deviations (n = 3) and were analyzed with the GraphPad Prims 7 software using variance analysis (one-way ANOVA) followed by Dunnett’s test with p = 0.0332, 0.0021, 0.002 and 0.0001 (one to four stars respectively). In figure below, a and b) cell viability of A549 treated with increasing concentration of silica in DMEM without and with serum, respectively. c) Cell viability of A549 treated with an increasing concentration of Curosurf in serum-free medium. d) Cell viability of A549 treated with an increasing concentration of silica-Curosurf dispersion at X = 20 in serum-free medium.


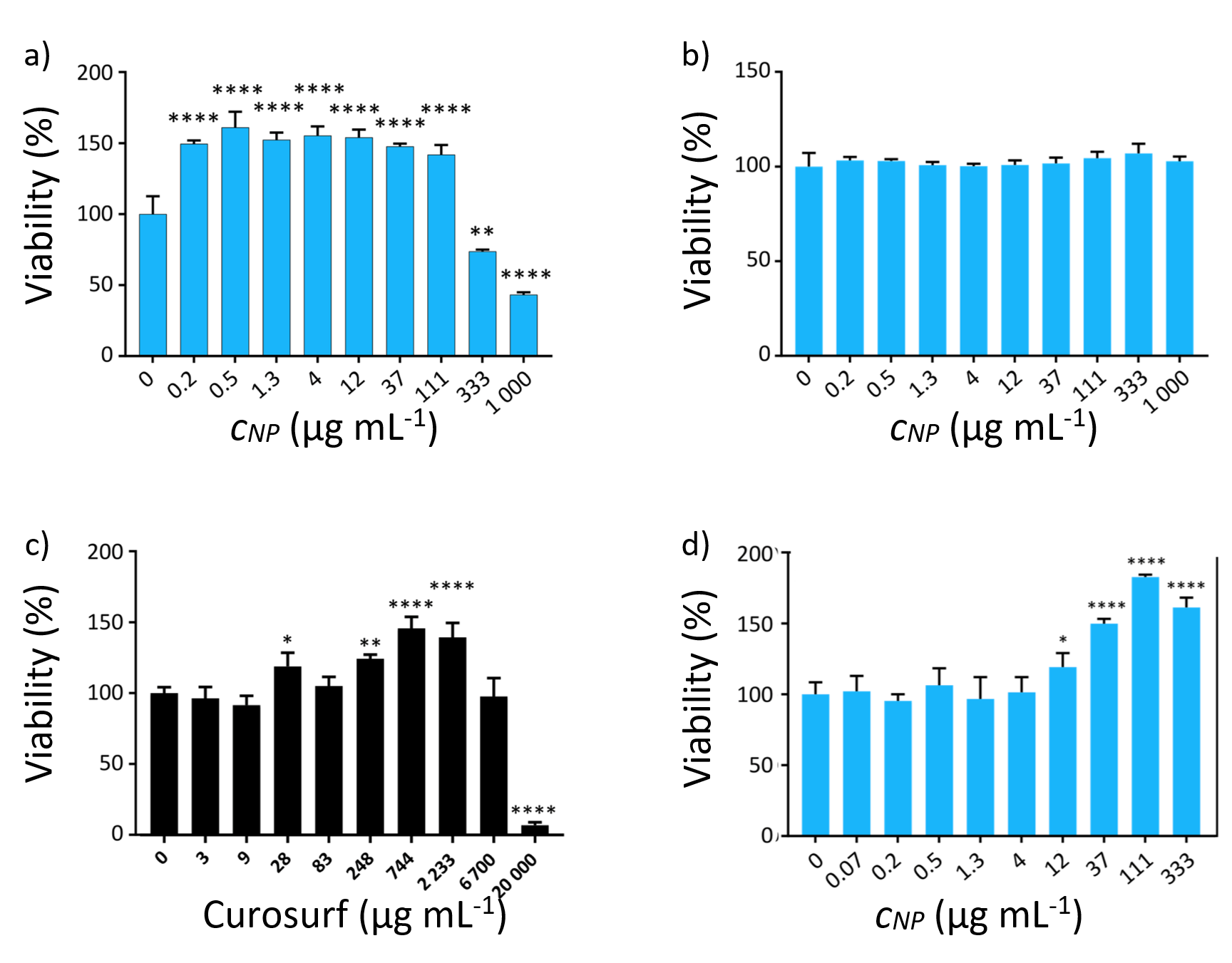


**Supplementary Information S10**

TEM of internalized silica nanoparticles without or with Curosurf

The figure below shows TEM images of positively charged silica internalized by A549 cells where vacuoles are also formed in the process. The nanoparticles enter the cells by disrupting the plasma membrane.


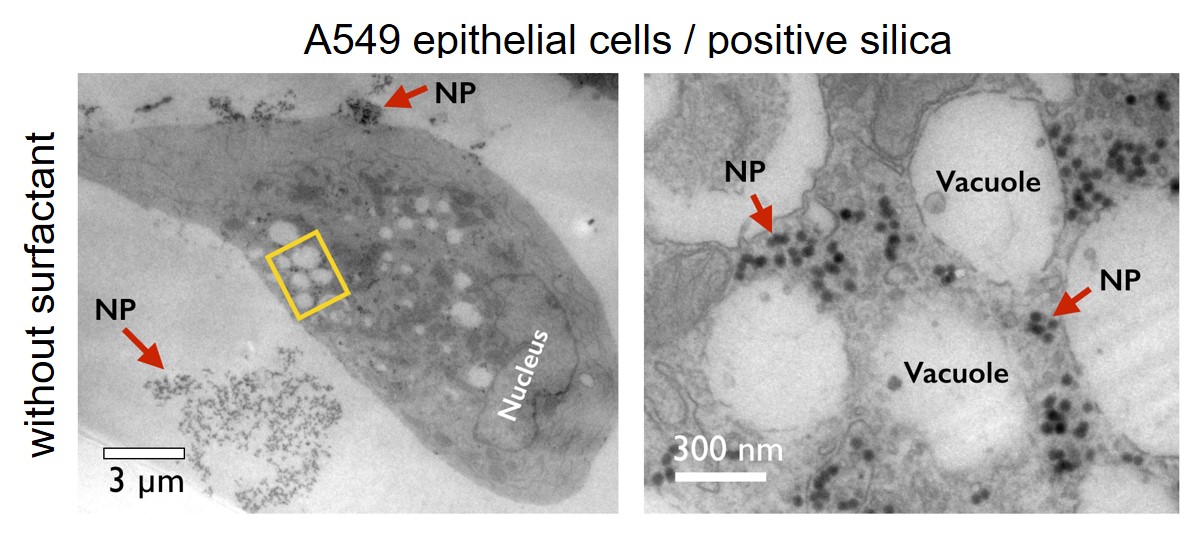


The figure below shows TEM images of supported lipid bilayer (SLB) coated positively charged silica internalized by A549 cells. Vacuoles are absent in this process. The nanoparticles are internalized via membrane bound vesicles.


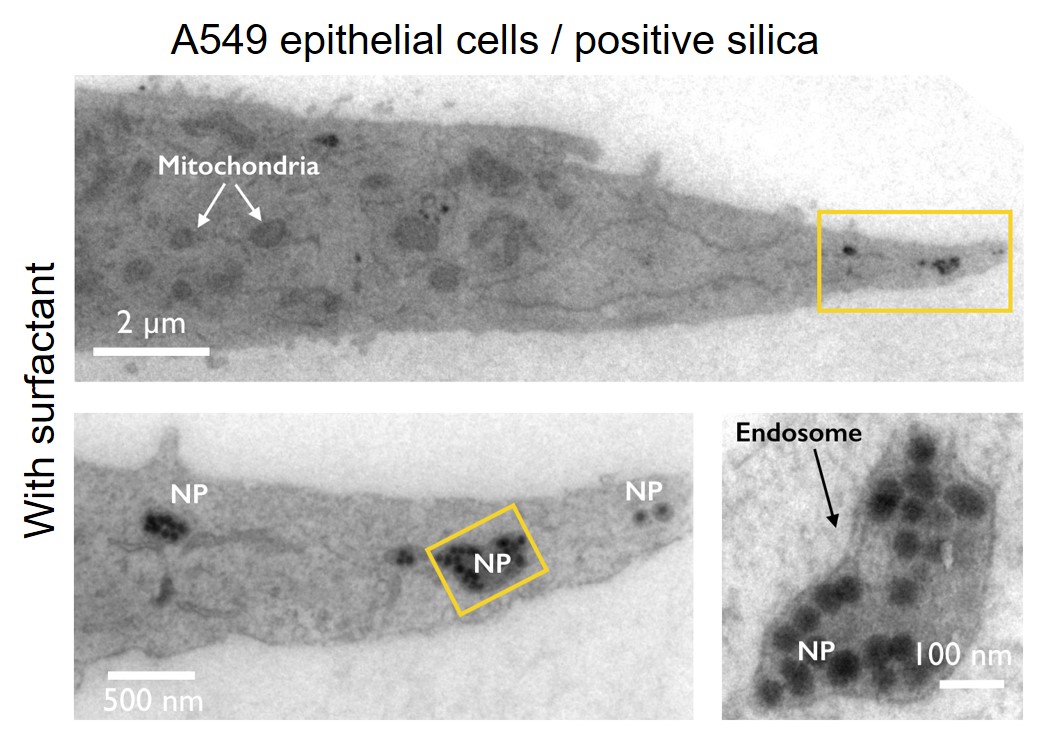


**References**

1. Braun, A.; Stenger, P. C.; Warriner, H. E., et al., A Freeze-Fracture Transmission Electron Microscopy and Small Angle X-Ray Diffraction Study of the Effects of Albumin, Serum, and Polymers on Clinical Lung Surfactant Microstructure. Biophysical Journal **2007**, 93 (1), 123-139.

2. Mousseau, F.; Berret, J. F., The role of surface charge in the interaction of nanoparticles with model pulmonary surfactants. Soft Matter **2018**, 14 (28), 5764-5774.

3. Mousseau, F.; Le Borgne, R.; Seyrek, E., et al., Biophysicochemical Interaction of a Clinical Pulmonary Surfactant with Nanoalumina. Langmuir **2015**, 31 (26), 7346-7354.

4. Mousseau, F.; Puisney, C.; Mornet, S., et al., Supported Pulmonary Surfactant Bilayers on Silica Nanoparticles: Formulation, Stability and Impact on Lung Epithelial Cells. Nanoscale **2017**, 9 (39), 14967-14978.

5. The Global Market for Aluminium Oxide Nanoparticles, in TECHNOLOGY REPORT No. 76. 2013, Future Markets, Inc.
